# Contrasting multistage and computer-based testing: score accuracy and aberrant responding

**DOI:** 10.3389/fpsyg.2023.1288177

**Published:** 2023-12-05

**Authors:** Georgios Sideridis, Hanan Ghamdi, Omar Zamil

**Affiliations:** ^1^Boston Children’s Hospital, Harvard Medical School, Boston, MA, United States; ^2^Department of Primary Education, National and Kapodistrian University of Athens, Athens, Greece; ^3^Education and Training Evaluation Commission, Riyadh, Saudi Arabia

**Keywords:** multistage testing, item response theory, person fit statistics, aberrant responding, guessing, carelessness

## Abstract

The goal of the present study was to compare and contrast the efficacy of a multistage testing (MST) design using three paths compared to a traditional computer-based testing (CBT) approach involving items across all ability levels. Participants were *n* = 627 individuals who were subjected to both a computer-based testing (CBT) instrument and a measure constructed using multistage testing to route individuals of low, middle, and high ability to content that was respective to their ability level. Comparisons between the medium of testing involved person ability accuracy estimates and evaluation of aberrant responding. The results indicated that MST assessments deviated markedly from CBT assessments, especially for low- and high-ability individuals. Test score accuracy was higher overall in MST compared to CBT, although error of measurement was enhanced for high-ability individuals during MST compared to CBT. Evaluating response patterns indicated significant amounts of Guttman-related errors during CBT compared to MST using person-fit aberrant response indicators. It was concluded that MST is associated with significant benefits compared to CBT.

## 1 Introduction

The validity of an individual’s results on national and international evaluations has a big impact on their life. Examples of invalid ability inferences that lead to lower than true ability include being placed in special education settings, being denied college admission, missing out on financial aid opportunities, stigma, and adverse emotional effects such as loss of self-esteem, having few professional opportunities, and having fewer financial benefits, etc. (Seligman, 1972; Abramson et al., 1989). Given these important implications, it is crucial that educational testing provide accurate conclusions about a person’s skills and competencies. Advancements in educational testing involve moving from paper and pencil to computerized assessments, engaging, computerized adaptive testing (Thompson and Weiss, 2011), or multistage testing (Wentzel et al., 2014; Zenisky and Hambleton, 2014).

There are two distinct categories of adaptive tests: computerized adaptive testing (CAT), which is the most prevalent and widely used, and multistage testing (MST). Adaptive tests use an algorithmic technique to adjust the degree of test difficulty in accordance with the examinee’s ability level, as determined by their performance over the duration of the test. One notable distinction between the two types of adaptive tests is in the method used. In computerized adaptive testing (CAT), the algorithm operates at the item level, focusing on the examinee’s performance on individual items. Consequently, the selection of the subsequent question is contingent upon the examinee’s performance on the preceding item. This implies that CAT exhibits many adaptive points at the item level, thus classifying it as an item-level adaptive test. The length of CAT may either be fixed or changeable. Therefore, the administration of the test concludes when the ability estimate reaches a threshold of precision beyond which there is no more alteration in the estimate due to the examinee’s performance, and ability converges when the level of standard error reaches a predetermined low cutoff value. Regarding multistage testing (MST), algorithms are used to analyze the performance of the examinee at certain stages, which consist of a collection of questions (also termed testlets). These adaptive points are found at both the stage level and between stages, as shown by Kim and Moses (2014) and Yan et al. (2014). In the present study, we will focus on evaluating multistage testing compared to traditional computer-based testing.

Studies contrasting CAT and MST have concluded that CAT may be advantageous to the measurement of extreme ability levels, low or high (Steinfeld and Robitzsch, 2021). It is more sensitive and granular as levels of ability are updated continuously (van der Linden and Glas, 2010). On the other hand, content balance and test security may be easier to achieve in MST designs compared to CAT (van der Linden, 2010). The complexity of CAT when selecting algorithms and the potential lack of control in item exposure may make MST more desirable (Pohl, 2014; Kim and Yoo, 2023) given that MST is a form of adaptive testing. To its advantage is also the fact that examinees can review and alter their earlier responses, providing a practical advantage over CAT designs (Parshall et al., 2002; Jodoin et al., 2006; Zwitser and Maris, 2015).

### 1.1 What is multistage testing and why is it potentially valuable?

MST is an adaptive test where sets of items are administered adaptively that consist of several sequential stages; each stage contains multiple units of different difficulty levels (i.e., easy, medium, hard) that represent the contents. The purpose of an adaptation procedure is to achieve the most precise estimates of a person’s proficiency in the shortest amount of time. Therefore, MST aims to reduce errors in estimating item parameters and ability levels and also the length of the test (Glas, 1988; Zheng and Chang, 2015). Multistage testing has recently undergone increased adoption as an alternative to both the classical linear test (CLT) and CAT (Kim et al., 2013). A multistage test is designed by selecting items from a pool that was calibrated before the test was administered previously, which benefits both developers and examinees; MST gives test developers more control over content balancing, item difficulty’s distribution, the quality of the test structure, dependencies among the items, and the distribution of non-statistical properties of the items such as the cognitive level. Adaptive multistage testing also allows examinees to review their responses within each module, while this is not available in CAT. Multistage testing involves the assembly of a test using several stages (most often 2–3). In the first stage, participants are administered a set of items, and their ability level is evaluated using number-correct (NC) or item response (IR) methodologies (Hendrickson, 2007). The latter engages maximum likelihood (MLE) or expected *a posteriori* (EAP) estimation procedure [10]. Based on performance during the first stage, a participant is” routed” to a module that is closer to their ability level in the second stage of a panel design (Kim et al., 2013). This procedure is followed in several stages until all modules are administered within a design.

Multistage testing is beneficial because it makes it possible to assess student success more effectively and precisely (Han and Guo, 2014; Han, 2020). The exam may be tailored to each test taker individually by employing a multistage test design, giving them the items that are most suited for their level of skill. Since subjects are exposed to material that is most suited for their skill level, this approach not only shortens tests but also probably improves measurement accuracy. By shortening the exam, weariness and overloaded cognitive attention processes may be overcome. The added burden of longer examinations includes, among other things, an increase in anxiety, disengagement, and withdrawal, the adoption of ineffective strategies such as guessing, and an increase in carelessness. The MST framework probably results in a more satisfying testing experience. In general, multistage testing is an effective technique for measuring educational outcomes because it enables a more accurate and efficient evaluation of student success. MST’s drawbacks include potential system complexity, administration and scoring challenges, increased costs, choosing the best algorithms and selection criteria for participant routing and testlet estimation, taking into account population diversity, controlling for item exposure rates, and the need for knowledgeable staff and administrator training.

The goal of the present study was to compare and contrast the efficacy of an MST design using three paths compared to a traditional computer-based testing approach involving items across all ability levels. Efficacy was judged by testing the accuracy with which theta scores were estimated and by using several person-fit indicators of aberrant response patterns. The examination of aberrant responding has been implemented for several reasons. First, aberrant responding reflects the unreliability and validity of the person responding; thus, it jeopardizes the inferences drawn for a person’s skills and competencies. The measurement of aberrant responding usually involves the examination of response vectors by evaluating observed versus expected patterns of behavior, such as the commonly accepted Guttman behavioral pattern, which posits that success diminishes as item difficulty increases. The use of various person-fit indices provides the advantage of examining aberrance due to various factors such as cheating, random responding, lack of motivation, misunderstanding instructions, successful guessing, and carelessness, etc. (Meijer and Sijtsma, 2001; Wollack, 2003; van der Linden and Guo, 2008).

## 2 Method

### 2.1 Participants and procedures

Data came from the assessment of mathematical competency using a 44-item unidimensional structure (CBT). This measure was given in full to a sample of participants, and then, the same participants were also subjected to a similar 44-item measure from which 22 items were common, and the remaining 22 items were tailed to three ability levels. That is, individuals whose ability was low were provided with content that was easier (easy module), participants who were of medium ability were administered items close to that ability lever (medium-difficulty module), and lastly, high-ability individuals were provided with items that were challenging and close to their skill level (difficult module). The total sample size was *n* = 627. The sample sizes per track were *n* = 210 for the Easy–Easy track, *n* = 281 for the Medium–Medium track, and *n* = 136 for the Difficult–Difficult track. The cutoff points to assign individuals to different ability levels were on pilot testing and stakeholder decision. Specifically, they were 0.30, 0.58, and 0.84 for the easy, medium-difficulty, and difficult tests, using the delta scoring metric (which ranges between 0 and 1); see Dimitrov (2018).

### 2.2 Measure

The General Ability Test (GAT) measures two general domains, namely, quantitative ability and verbal ability. Each domain encompasses subdomains. For example, the quantitative ability domain assesses arithmetic, number sequence, analysis, logic, inductive reasoning, spatial ability relations, and visualization. For verbal ability, the subdomains include antonyms, sentence completion, and reading comprehension. Furthermore, the two dimensions were considered to be unidimensional. Students who are willing to enroll in universities and colleges in Saudi Arabia should take the GAT, which is considered the main administration requirement. In the present study, for the evaluation of multistage testing, only the quantitative domain was utilized, which comprises 44 items.

### 2.3 Data analyses

#### 2.3.1 Measurement accuracy and error of measurement

Estimation of model fit in both conditions was assessed using item response theory (IRT) models, specifically the 2PL model, which models both item discrimination and item difficulty parameters. Evaluative criteria involved the chi-square statistics, the root mean square error of approximation (RMSEA), and two descriptive fit indices, namely, the CFI and TLI. Following model fit, theta scores were estimated per person, which in the case of MST reflected a model with all items and concurrent calibration using the 22 common items. Along with estimates of theta, standard errors were also computed. Further analyses involved person statistics and visual means that are described below. All analyses were conducted using Mplus 8.9 and the Perfit package in R.

Power for the unidimensional item response model was tested using the procedure put forth by MacCallum et al. (1996). The procedure involves estimating the power to select a well-fitted model (i.e., with RMSEA = 0.05) over an unacceptable model (i.e., with RMSEA = 0.08), as a function of the difference in estimated model parameters, and estimating the non-centrality parameter. Using an alpha level equal to 5% and the smallest sample size (i.e., *n* = 136) of the sample size involved in the Difficult-Difficult track, the results indicated that power to detect was significant, and the difference in the RMSEA values was over 98%. Thus, there were ample levels of power in estimating the item response models.

#### 2.3.2 Person aberrant responding patterns

There are a large number of studies examining aberrant responding using the IRT framework and using as a basis the Guttman scaling pattern (Meijer, 1996; Meijer and Sijtsma, 2001). Based on that pattern, there is the expectation that individuals of medium ability will be successful in the easy tasks and correspondingly unsuccessful in the difficult tasks, whereas for tasks close to their ability level, success rates are expected to be approximately 50%. Two major studies have evaluated more than 40 such indicators of aberrance (Meijer and Sijtsma, 2001) using Monte Carlo simulations (Karabatsos, 2003). In the present study, we selected four such indices that were found to behave in acceptable ways as a means to identify careless responding, guessing, and/or cheating. These are briefly described next.

The *number of response vector errors* reflecting the Guttman pattern (Guttman, 1944) is estimated using the G index as follows:


(Equation 1) $$G=\underset{h,e}{\sum }X_{nh}\left(1-X_{ne}\right)$$

Large values are indicative of aberrant responding, being suggestive of random responding, carelessness, or inattention. However, because the index is not normed, van der Flier (1977) proposed the normed index, as shown in Equation 2, which is standardized for the instrument’s length:


(Equation 2) $$\mathrm{Gnorme}d^{*}=G/r_{n}\left(L-r_{n}\right)$$

Another index proposed by the same author is *U3* (van der Flier, 1977; see also Emons et al., 2005):


(Equation 3) $$U3=\frac{𝓁n0\;\left(X_{n}^{*}\right)-𝓁n\left(X_{n,}\right)\;}{𝓁n\left(X_{n}^{*}\right)-\left(X_{n}^{\prime }\right)}$$

This estimates the Guttman pattern with a specific set of weights wg = ln (πg/1–πg). Large values are again indicative of aberrant responding in the form of carelessness, inattention, lack of motivation, guessing, or randomness. Simulation studies indicated the excellent efficiency of U3 to accurately assess random responding (Karabatsos, 2003; Beck et al., 2019).

The *Norm Conformity Index (NCI)* (Tatsuoka and Tatsuoka, 1982, 1983) is a standardized index, linearly related to G, which reflects a Guttman pattern in its maximum score of unity, with zero representing a reversed Guttman pattern. Given its relationship to G, it can be estimated as follows:


(Equation 4) NCI=1−2∗GNormed

With low values in NCI being indicative of aberrant responding.

## 3 Results

### 3.1 Psychometrics of measure

A 2PL model was fit to the data, and model fit was evaluated using global fit indices such as the chi-square test, descriptive fit indices such as the CFI, and residuals, namely, the root mean square error of approximation (RMSEA). The 44-item unidimensional CBT quantitative measure pointed to a modest model fit with significant discrimination parameters ranging between 0.20 and 0.68. The RMSEA was 0.038 with a 95% confidence interval ranging between 0.035 and 0.041, which is acceptable. The chi-square test was significant but was not valued heavily as it is an indicator of exact fit. Last, estimates of the fit indices were CFI = 0.798 and TLI = 0.788, which are on the low side. The respective estimates for the MST measure were as follows: the Easy–Easy track (RMSEA = 0.030, RMSEA.C.I._95%_ = 0.013–0.042, CFI = 0.751, TLI = 0.727) had discrimination parameters ranging between 0.10 and 0.58; the Medium–Medium track (RMSEA = 0.019, RMSEA.C.I._95%_ = 0.000–0.030, CFI = 0.839, TLI = 0.826) had discrimination parameters ranging between 0.10 and 0.67; and the Difficult–Difficult track (RMSEA = 0.029, RMSEA.C.I._95%_ = 0.009–0.041, CFI = 0.804, TLI = 0.792) had discrimination parameters ranging between 0.15 and 1.24. After saving theta estimates, the results confirmed the hypothesized functioning of the tracks, with participants in the EE track having the lowest ability, participants in the medium track having mid-level ability, and participants in the DD track having the highest level of ability.

### 3.2 MST and CBT differences in theta and conditional standard errors of measurement

**Figure 1:**
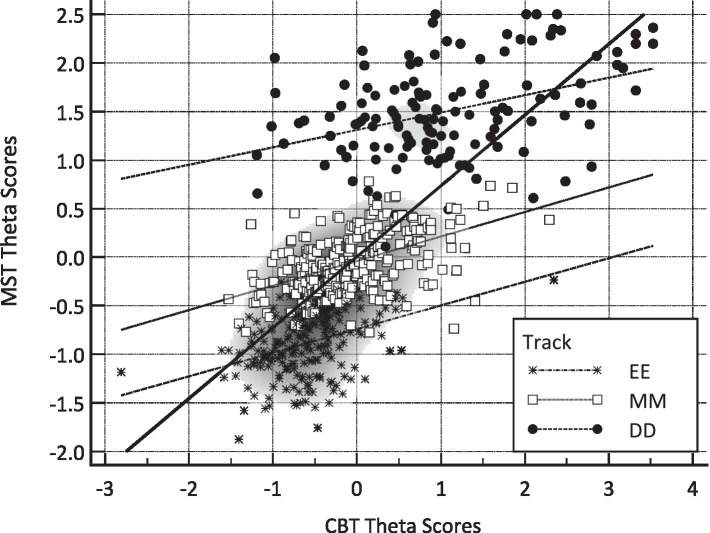
Scatterplot displaying the relationship between MST (vertical axis) and CBT (horizontal axis) factor scores. EE, Easy–Easy track, MM, Medium–Medium track, DD, Difficult–Difficult track.

Two types of analyses were involved in evaluating the two conditions under which ability and their corresponding error were estimated, namely, a correlational analysis by engaging scatterplots and prediction lines and a mean level analysis using analyses of variance (ANOVAs). Figure 1 displays scatterplots for theta estimates between the MST (vertical axis) and CBT (horizontal axis) conditions, with linear predictive lines fitted separately per track (using dotted prediction lines) and overall (using the solid predictive line). As shown in the figure, prediction slopes were almost parallel, reflecting similar magnitude relationships across the tracks. When contrasting MST and CBT theta estimates, correlations were *r* = 0.377 for the EE track, *r* = 0.496 for the MM track, and *r* = 0.388 for the DD track. Therefore, the greatest similarity between the estimates obtained from the Multistage Test (MST) and the Computer-Based Test (CBT) was seen in relation to persons with moderate abilities. In contrast, the theta scores of individuals with lower and higher abilities showed little similarity across the different testing conditions.

**Figure 2:**
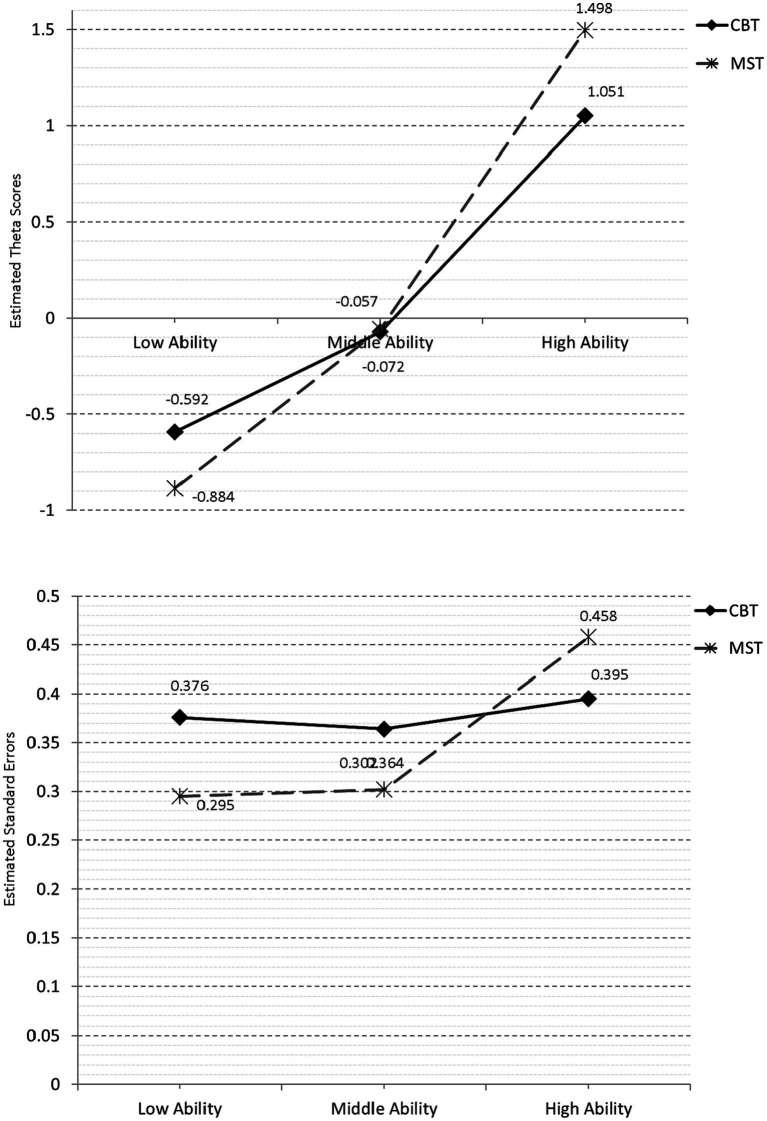
Theta estimates by track and condition (upper panel) and standard errors (lower panel).

Differences in level were estimated using 2×3 within/between analyses of variance, with the three tracks being the between-groups condition and CBT vs. MST the within groups condition There was a significant main effect for condition [*F*(1, 624) = 4.405, *p* = 0.036], with the mean theta scores being significantly higher in the MST condition (M_Theta_ = 0.186) compared to the CBT condition (M_Theta_ = 0.129). However, a significant interaction was also evident [*F*(2, 624) = 53.920, *p* < 0.001] (see Figure 2, upper panel), suggesting that the CBT condition individuals of low ability had significantly higher scores compared to the MST condition and that the opposite was true for high-ability individuals whose scores were significantly higher during the MST condition.

The same 2×3 design was applied to the assessment of conditional standard errors of measurement. The results pointed to a significant main effect for condition [*F*(1, 624) = 271.678, *p* < 0.001] and a significant interaction [*F*(2, 624) = 652.228, *p* < 0.001]. As shown in Figure 2 (lower panel), the significant interaction pointed to significantly lower amounts of error for the low- and mid-ability groups but significantly elevated errors for the high-ability individuals in the MST condition.

### 3.3 Contrasting MST and CBT designs using person aberrant response patterns

Table 1 displays the results from contrasting the CBT and MST measures across tracks using four-person indicators of aberrant responding. All indicators favored the MST condition compared to CBT. Small-to-medium-sized differences (Cohen, 1992) were revealed for the MM path, which targeted individuals of moderate ability. Thus, differences for this group were least pronounced albeit significantly different from zero. The largest differences were observed for low-ability (EE path) and high-ability (DD) individuals. For example, based on the G index, the number of Guttman errors for the low-ability group was 144 in the CBT compared to 107 in the MST condition. This effect was augmented in the high-ability group for which the mean number of Guttman errors was 118 in CBT compared to 41 during the MST condition, reflecting a reduction in errors of 65%.

**Table 1:** Differences between CBT and MST designs on person-fit statistics per module and path.

Person-fit index	CBT Mean/SD	MST Mean/SD	*T*-test	Cohen’s D E.S.	Effect size convention^†^	Conclusion: favoring
Path 1 (Easy–Easy) (*n* = 210)						
1. G	143.70/41.350	107.81/34.23	4.696*	0.95	Greater than Large	MST
2. G_normed_	0.362/0.098	0.309/0.104	9.680*	0.53	Medium to Large	MST
3. U3	0.376/0.111	0.312/0.107	6.002*	0.59	Medium to Large	MST
4. NCI	0.275/0.197	0.383/0.209	5.419*	0.53	Medium to large	MST
Path 6 (Medium–Medium) (*n* = 281)						
1. G	117.93/35.060	109.85/34.12	2.769*	0.23	Small to Medium	MST
2. G_normed_	0.324/0.091	0.303/0.091	2.814*	0.24	Small to Medium	MST
3. U3	0.289/0.096	0.260/0.096	3.575*	0.30	Small to Medium	MST
4. NCI	0.351/0.182	0.394/0.182	2.814*	0.24	Small to Medium	MST
Path 11 (Difficult–Difficult) (*n* = 134)						
1. G	118.34/44.806	40.86/27.96	16.913*	2.10	Greater than Large	MST
2. G_normed_	0.305/0.104	0.241/0.121	4.623*	0.57	Medium to Large	MST
3. U3	0.279/0.096	0.218/0.105	5.005*	0.62	Medium to Large	MST
4. NCI	0.389/0.208	0.518/0.242	4.623*	0.57	Medium to Large	MST

*p < 0.05. *T*-test values are shown in absolute terms. Effect size conventions are S, small, M, medium, and L, Large, with estimates within two levels being termed as a range.

^†^As per Cohen (1992) conventions on effect size.

**Figure 3:**
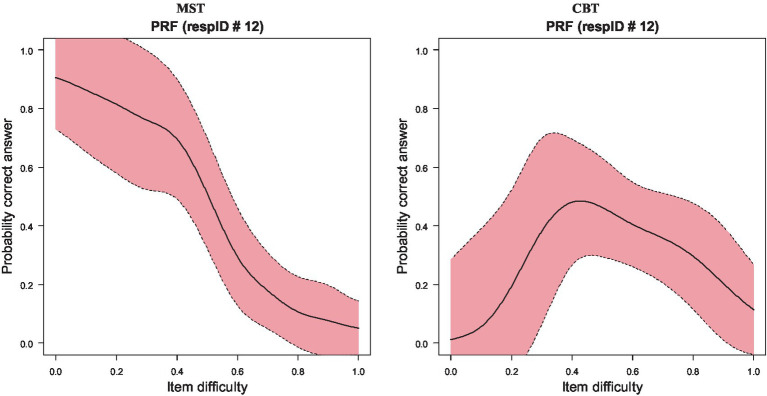
Person response functions (PRFs) for participant no. 12 when evaluated using MST (left) and CBT (right) measurements.

An example of the differences between MST and CBT is illustrated with a single participant (see Figure 3). Participant Number 12 had a theta estimate of −1.111; thus, this participant was a low-ability person. When participant 12 was evaluated using the EE track, their person response curve was depicted with a downward trend, in that as item difficulty increased, the probability of a correct response was decreased (see Figure 3, left panel). When the same participant was evaluated using traditional testing in computerized form, their ability was evaluated at −0.185 logits, classifying them slightly below the mean of theta or, in other words, of almost average ability. The difference between the two measurements was almost one logit, which is quite large. Furthermore, when plotting their successes over item difficulties, the person response curve reflected an unexpected pattern in that their probability of success was at maximum levels with medium-difficulty items, but the respective probabilities for easier items were much lower, possibly reflecting random responding at the onset of the test or careless responding (see Figure 3, right panel). This person emitted 199 Guttman errors in responding during the CBT condition, with the respective estimates during MST being 97, which is less than half. These findings highlight the appropriateness of the MST assessment for this participant compared to CBT.

An ancillary analysis was undertaken to test the hypothesis that the differences in theta scores observed in the DD track were a function of aberrant responding patterns. In other words, the large deviations between person estimates in the two conditions reflected full error estimates in the CBT measurement compared to MST for the same individuals. For that purpose, individuals with a difference score from CBT to MST of greater than or equal to 1 logit were selected to reflect large differences in ability of the same participants across measurements. During the DD condition, i.e., for the high-ability group, there were 53 such participants. Out of the *n* = 53 participants, 14 had lower scores in MST compared to CBT, and *n* = 39 had higher estimated theta scores. Given the within-person design, *t*-tests were implemented to evaluate potential differences in the amount of Guttman errors for those individuals whose scores were saliently different from CBT to MST (by = > ±1 logit). When contrasting positively changed scores, no significant differences were observed in the amount of Guttman errors using the G index. When contrasting negatively changed scores, in that participants during MST were estimated to have higher ability compared to CBT, results indicated significant effects. Specifically, the number of Guttman errors for those participants was 133.5 during the CBT condition and 93.4 during the MST condition. Thus, the observed inflation of theta scores during the DD condition for able participants may likely be attributed to the less precise measurement that took place for these participants during the CBT measurement as aberrant responding was highly prevalent for these participants. In terms of effect size, the difference in the number of errors was 0.99 of a standard deviation, reflecting an effect larger than large, based on Cohen (1992) conventions on what constitutes small–medium–large effects.

## 4 Discussion and concluding remarks

The objective of the current research was to examine and analyze the effectiveness of a multi-stage test (MST) design that utilizes three different pathways in comparison to a conventional test that includes questions spanning all levels of ability. The evaluative criteria involved the psychometrics of the measure, theta estimation precision, and the presence of aberrant response patterns.

The most important finding was that measurement using MST was superior compared to traditional computer-based measurement. Overall, the measured instrument was functioning in better ways using unidimensional-related indices, and aberrance was more prevalent during CBT compared to MST. This finding is in agreement with past studies in which enhanced accuracy when employing MST compared to traditional testing was observed (Wang et al., 2012). However, what is far more interesting is that a significant reduction in measurement error has important implications for test length. That is, with a more precise estimation in theta, fewer items would be required before a person’s score would converge within computerized adaptive testing (Han, 2020). Thus, the benefits in time, effort, and cost are significantly reduced as score precision becomes elevated.

A second important finding was that significant divergence between theta scores was observed in low-ability and high-ability individuals for whom usually the error is enhanced, compared to medium levels of ability for which there was a striking resemblance between the MST and CBT conditions. This finding agrees with the simulation study of Svetina et al. (2019) in which item difficulty rates were recovered most precisely in items of moderate difficulty and less so for easy and difficult items. Interestingly, although the overall error estimate favored the MST condition, the amount of error was higher for the high-ability group. We can only speculate why this is the case, but the modest sample size (*n* = 134) may be accountable for that effect.

A third important finding was that our hypothesis that divergent theta estimates between conditions may be linked to aberrant response patterns was also verified for high-ability individuals. Specifically, the number of Guttman-related errors was significantly higher for high-ability individuals during the CBT testing compared to MST testing, reflecting an effect size of a 1 standard deviation. Whether the aberrance during CBT was due to fatigue, less sensitive content for that ability group, or the operation of psychological processes that inhibit achievement remains to be studied in the future.

There are various limitations associated with the current investigation. The sample size in the EE and DD tracks was small due to restrictions posed by the within-person design employed in the current investigation. Therefore, it is not justified to make judgments about the generalizability of the results to the population. In addition, the person-fit indicators included in this study capture only a limited range of deviant behaviors. The potential occurrence of atypical responses during the computer-based measurement for various forms of deviant behavior cannot be assumed and should be examined in future research endeavors.

### 4.1 Future directions

It will be critical in the future to assess MST characteristics that might result in better measurements. For instance, a lot of brief modules boost measurement accuracy (Zheng and Chang, 2011). The effectiveness and applicability of MST testing in comparison to traditional testing will also be aided by choosing the most suitable MST design in terms of the number of modules/tracks and/or the use of testlets and by evaluating the routing strategy employed (Wentzel et al., 2014; Zheng et al., 2014; Svetina et al., 2019). Furthermore, the use of mixture modeling may aid evaluation of the participant performance in the tracks and add conclusions regarding the differentiation of tracks, given the evidence for latent class homogeneity and separation. Last, as the results of the present study have mostly been equivocal, comparisons between the effectiveness of MST and CAT are required. However, the results of this investigation support the assertion that MST increases measurement accuracy and precision when compared to conventional testing.

## Data availability statement

The raw data supporting the conclusions of this article will be made available by the authors, without undue reservation.

## Ethics statement

The studies involving humans were approved by Education and Training Evaluation Commission ethics committee. The studies were conducted in accordance with the local legislation and institutional requirements. The participants provided their written informed consent to participate in this study. Written informed consent was obtained from the individual(s) for the publication of any potentially identifiable images or data included in this article.

## Author contributions

GS: Conceptualization, Formal analysis, Methodology, Writing – original draft, Writing – review & editing. HG: Formal analysis, Funding acquisition, Methodology, Writing – review & editing. OZ: Data curation, Formal analysis, Methodology, Software, Writing – review & editing.
